# Detection of Specific Building in Remote Sensing Images Using a Novel YOLO-S-CIOU Model. Case: Gas Station Identification

**DOI:** 10.3390/s21041375

**Published:** 2021-02-16

**Authors:** Jinfeng Gao, Yu Chen, Yongming Wei, Jiannan Li

**Affiliations:** 1 Aerospace Information Research Institute, Chinese Academy of Sciences, Beijing 100094, China; gaojf@radi.ac.cn (J.G.); chenyu@radi.ac.cn (Y.C.); lijiannan19@mails.ucas.ac.cn (J.L.); 2 College of Resources and Environment, University of Chinese Academy of Sciences, Beijing 100049, China

**Keywords:** YOLO-S-CIOU, SE-ResNeXt, CIoU loss, gas station, object detection, remote sensing image, specific building

## Abstract

The specific building is of great significance in smart city planning, management practices, or even military use. However, traditional classification or target identification methods are difficult to distinguish different type of buildings from remote sensing images, because the characteristics of the environmental landscape around the buildings (like the pixels of the road and parking area) are complex, and it is difficult to define them with simple rules. Convolution neural networks (CNNs) have a strong capacity to mine information from the spatial context and have been used in many tasks of image processing. Here, we developed a novel CNN model named YOLO-S-CIOU, which was improved based on YOLOv3 for specific building detection in two aspects: (1) module Darknet53 in YOLOv3 was replaced with SRXnet (constructed by superimposing multiple SE-ResNeXt) to significantly improve the feature learning ability of YOLO-S-CIOU while maintaining the similar complexity as YOLOv3; (2) Complete-IoU Loss (CIoU Loss) was used to obtain a better regression for the bounding box. We took the gas station as an example. The experimental results on the self-made gas station dataset (GS dataset) showed YOLO-S-CIOU achieved an average precision (AP) of 97.62%, an F1 score of 97.50%, and had 59,065,366 parameters. Compared with YOLOv3, YOLO-S-CIOU reduced the parameters’ number by 2,510,977 (about 4%) and improved the AP by 2.23% and the F1 score by 0.5%. Moreover, in gas stations detection in Tumshuk City and Yanti City, the recall (R) and precision (P) of YOLO-S-CIOU were 50% and 40% higher than those of YOLOv3, respectively. It showed that our proposed network had stronger robustness and higher detection ability in remote sensing image detection of different regions.

## 1. Introduction

The detection of specific buildings such as gas stations, schools, and airports are of great significance in smart city planning [1], management practices, or even military use. However, although the accuracy of specific buildings detection utilizing surveying and mapping is high, it is time-consuming and laborious. The renewal cycle is long, which cannot meet rapid updating and changing urban construction. With the rapid development of sensors and aerospace technology, the spatial resolution, temporal resolution, and spectral resolution of remote sensing images are getting higher and higher. Remote sensing images can be obtained in a short period of time and contain more detailed information, which makes it possible to detect a certain type of buildings from remote sensing images.

Traditionally, the specific buildings in remote sensing images are detected mainly based on artificial features, such as corners, edges, and textures. Zhang et al. [2] used the edge, texture, and color features to realize the remote sensing image’s airport detection. Zhong et al. [3] realized illegal building detection in unmanned aerial vehicle (UAV) images by using a vegetation index, morphological building index (MBI), texture feature, and spectral characteristics of red and green bands. Yu [4] classified the buildings into four categories: factory buildings, old fashioned dwellings, multistory residential buildings, and high-rise buildings by spectral location combined analysis (SLCA) and object scene correlation analysis (OSCA). Although these methods are easy to understand, these methods’ accuracy is low due to the limited amount of information and the lack of spatial structure information in the manual detection rules. Moreover, these methods’ poor transferability also makes them not widely used in various types of buildings.

Convolution neural networks (CNNs) have a strong capacity to mine information from the spatial context, and its automated learning mechanism allows accurate reusability in different objects. Therefore, CNN is widely used in remote sensing images for object detection [5,6,7]. Currently, region-based CNNs and regression-based CNNs are two kinds of mainstream CNNs for object detection. The region-based CNNs are two-step networks. They obtain the boundary box of the target first and then predict the target category. The step-by-step learning strategy makes the detection accuracy of this type of networks high, but they are time-consuming. Accordingly, it is hard for them to deal with numerous remote sensing images in real-time. The classic representatives of this type of networks are R-CNN [8], Fast R-CNN [9], Faster R-CNN [10], and Mask R-CNN [11]. The regression-based CNNs are one-step networks, which regard the whole prediction process as a regression process. The simplification of the process has brought about an increase in speed. YOLO (You Only Look Once) [12] series network is typical of regression-based CNNs. It has developed several versions, such as YOLO, YOLOv2 [13], YOLO9000 [13], and YOLOv3 [14]. Among these versions, YOLOv3 achieves better compromise for both speed and precision [15], which can satisfy the needs of real-time applications and generate excellent precision.

YOLOv3 is commonly applied in detecting various targets in remote sensing images because of its practicability. Yu et al. [16] performed rapid and accurate detection of airports in remote sensing images by combining YOLOv3, saliency detection, and connected region extraction. In post-earthquake high-resolution images, Ma et al. [17] realized the high-precision identification of collapsed buildings using an improved YOLOv3. Chen et al. [18] realized the ship target detection in a complex water surface environment through an improved YOLOv3. However, these studies have exposed the low recall rate and inaccurate bounding box of YOLOv3. Therefore, the learning ability and the prediction box regression method of YOLOv3 need to be further improved. Simultaneously, due to the limitation of hardware conditions in practical application, it is also essential to control the improved networks’ parameter complexity.

This study developed a novel CNN model named YOLO-S-CIOU (YOLO, S, and CIOU refer to YOLOv3, SRXnet, and CIoU loss, respectively), which was improved based on YOLOv3 for specific building detection. First, module SRXnet was constructed by repeating SE-ResNeXt [19]; then, module Darknet53 in YOLOv3 was replaced with SRXnet to significantly improve the feature learning ability of YOLO-S-CIOU while maintaining the similar complexity as YOLOv3; last, Complete-IoU Loss [20] (CIoU Loss) was used to obtain a better regression for the bounding box. We took the gas station as an example, which is less studied currently.

This paper’s remainder is structured as follows: The dataset production, model improvement, evaluation indicators, and experimental settings are provided in Section 2. Analysis and discussion of the experimental results are provided in Section 3. Section 4 finally summarizes the study.

## 2. Materials and Methods

### 2.1. Dataset

#### 2.1.1. Dataset Preparation

There is no high-quality gas station data that meet study needs in the current open source building data [21,22]. Thus, it becomes necessary to construct a new dataset, which contains high-quality gas station data and provides a good expansion to the current open-source building dataset. For the convenience of the quote, we named the self-made gas station dataset, the GS dataset.

**Figure 1:**
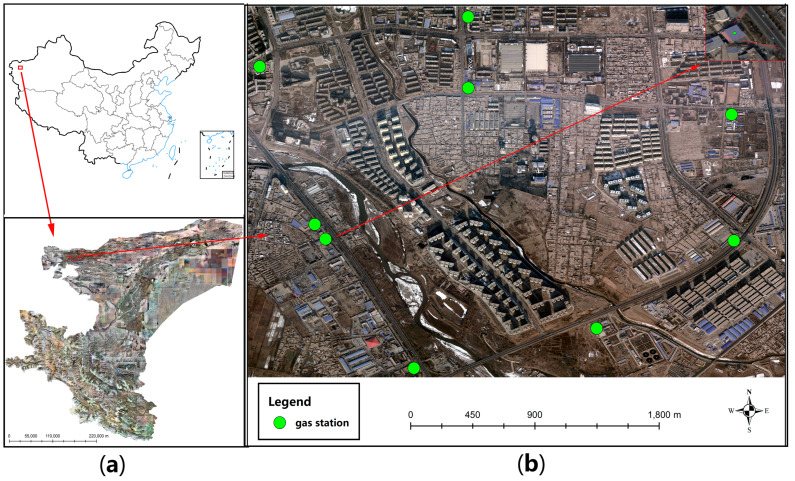
Part of the remote sensing images and POI data used in the creation of the gas station (GS) dataset: (**a**) thumbnail of remote sensing images of Kashgar area provided by the funded project and (**b**) a small piece of POI data samples used to assist the production of the GS dataset.

**Figure 2:**
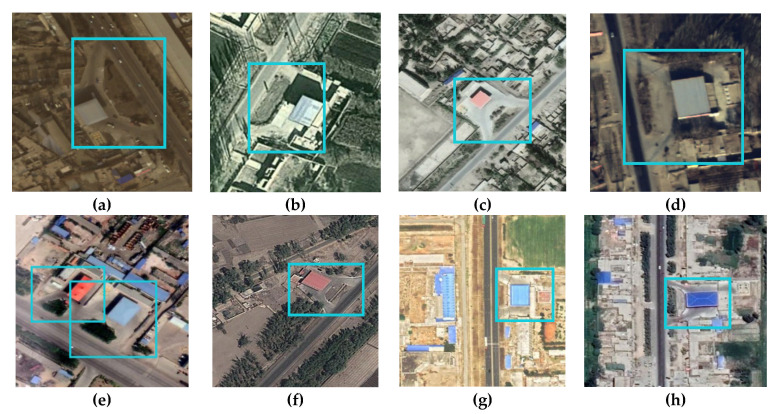
Examples of images and label frames of the GS dataset: (**a**–**d**) are gas station data with different image qualities from remote sensing images provided by the funded project and (**e**–**h**) are gas station data with varying image qualities from Google Maps. The blue boxes are the label frames.

During the production of GS dataset, we used aerial remote sensing images and WorldView images provided by the funded project that could cover the Kashgar area of Xinjiang Uygur Autonomous Region (as shown in Figure 1a), and we also used Google Maps that could cover Xinjiang Uygur Autonomous Region except for Kashgar area and Tumshuk City. Among them, the resolution of the aerial remote sensing images and WorldView images used was 0.5 m, and the resolution of Google Maps used was 0.45 m. The generation of the GS dataset combines the interpretation marks of gas stations on remote sensing images and combines the POI (Point of Interest) data collected by GPS to ensure the accuracy of the dataset. Some examples of POI data are shown in Figure 1b. Some targets occluded by trees or had low image quality were selectively excluded when the dataset was being produced, further ensuring the quality of the dataset. On remote sensing images, gas stations are often located close to highways. The overall shape of the entrance and exit access shapes like the Chinese character “eight”. The roof of gas stations is often blue or red, as shown in Figure 2a–h.

GS dataset contains 620 gas station pictures with a scale of 416 pixels × 416 pixels. Most of these pictures contain only one gas station. Only a few pictures contain two. After the image portion of the dataset had been made, Labelimg was used to mark the image. Figure 2 shows the image and label frame of the dataset.

#### 2.1.2. Data Enhancement

Deep learning networks often require a large amount of data to obtain satisfactory training results because of lots of parameters. Although the control of data quality is fully considered in the production process, the data quantity of the GS dataset is small. This results from the limited number of gas stations available for collection and the elimination of the data with poor quality during the production process. Besides, limited workforce and material resources also limit the scale of the dataset. Therefore, the study used data enhancement [23,24] to increase the data’s diversity and improve the model training result’s accuracy.

According to the needs of the experiment, the dataset was further subdivided into training, validation, and the testing sets. Numerous enhancement methods (cropping, rotation, flipping, noise increase, and color transformation) were used in the three sets under the target characteristics. The detailed values of each set after enhancement are listed in Table 1.

#### 2.1.3. Remote Sensing Images to Be Tested

**Figure 3:**
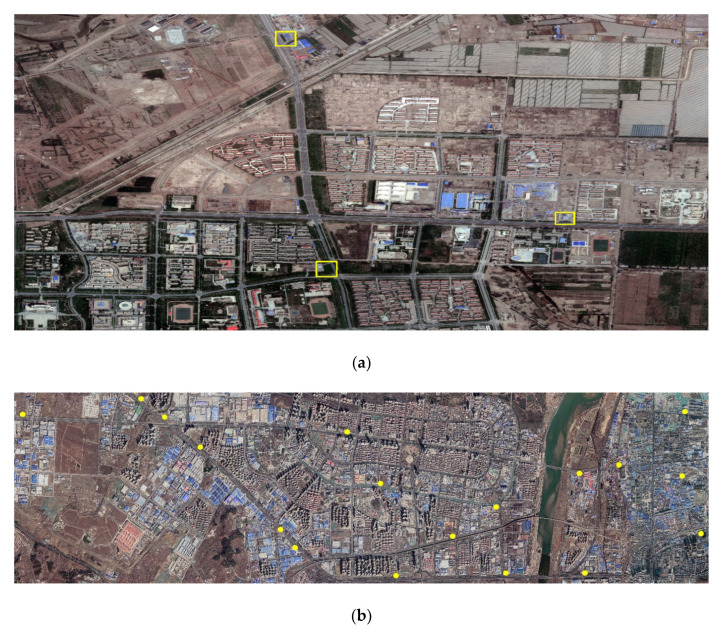
The images to be tested and the ground truth of gas stations. (**a**) WorldView data to be tested and (**b**) Google map data to be tested. Yellow squares and yellow points represent the ground truth of gas stations.

To test the detection effect of the CNN proposed in this study for remote sensing images, the WorldView data covering a small scope of Tumshuk City in the Xinjiang Uygur Autonomous Region and Google maps covering a small scope of Yantai City in Shandong Province were used to detect gas stations. It is worth noting that these images were not used in the production of GS dataset, ensuring the information contained in these images did not disclose to the CNN before testing. Figure 3 displays the images to be tested and the ground truth (yellow box and point) of gas stations that have been verified after in-person visits.

### 2.2. Method

To deal with the remote sensing image detection task and improve the low recall rate of classical YOLOv3, it is necessary to improve the learning ability of YOLOv3 further. Simultaneously, due to the limitation of hardware conditions in the practical application, the complexity of the improved model cannot be significantly increased. Therefore, we developed a novel CNN model named YOLO-S-CIOU, which was improved based on YOLOv3 for specific building detection. In the new CNN, cardinality was introduced, and useful features were better utilized through the addition of SEnet, both of which were realized by replacing the Darknet53 in YOLOv3 with SRXnet (constructed by SE-ResNeXt [19]). Additionally, the position loss of the loss function in YOLOv3 was replaced with CIoU loss [20]. In the bounding box regression, the overlap area, distance of central point, and the aspect ratio’s consistency are comprehensively considered to realize the fast and accurate regression of the prediction box.

#### 2.2.1. Structure of You Only Look Once Version 3 (YOLOv3)

The network proposed in the study is improved based on YOLOv3. As the third-generation of YOLO, YOLOv3 [14] inherited numerous features from the previous two generations of YOLO and introduced several innovations. It was primarily improved in three areas: improvement of the feature extraction network, introduction of the multiscale feature detection, and the use of logistic regression in the classifier.

**Figure 4:**
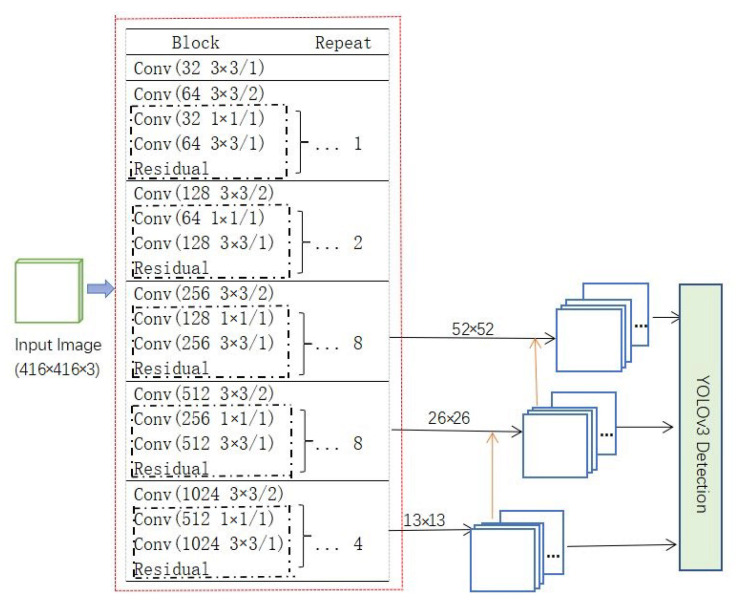
The architecture of YOLOv3. The red box part represents Darknet53 without fully connected layers, while the yellow arrow indicates upsampling. A convolution layer is denoted as (filters and filter size/step size).

The main body of YOLOv3 can be decomposed into two components: a feature extraction network and a multiscale prediction network. Unlike YOLOv2 [13], YOLOv3 uses Darknet53 as its feature extraction network. Besides, convolution with a step size of 2 was used to achieve downsampling in YOLOv3. The method of upsampling, similar to the feature pyramid network [25] (FPN), and fusion are used to realize the combination of deep features and superficial features, making good use of deep features. Nine prior frames obtained by the K-means algorithm were allocated to the scales of 13 ×13, 26 × 26, and 52 × 52. Finally, YOLOv3 uses logistic regression to predict the object category. In this way, YOLOv3 can support multilabel objects and greatly reduce the amount of computation required. The detailed structure of YOLOv3 is shown in Figure 4.

The loss function of CNN is vital. Based on the information obtained in the final prediction, YOLOv3′s loss function was divided into four parts: XY coordinate loss (XY_loss), length and width loss (WH_loss), confidence loss (Confidence_loss), and category loss (Class_loss). These losses were calculated separately according to their characteristics. Except for WH_loss, the other loss functions used binary cross-entropy.

Although YOLOv3 achieved remarkable accuracy and speed compared with other networks in the same period, it also had several shortcomings, such as poor positioning frame accuracy and low recall rate.

#### 2.2.2. SE-ResNeXt Network Structure

**Figure 5:**
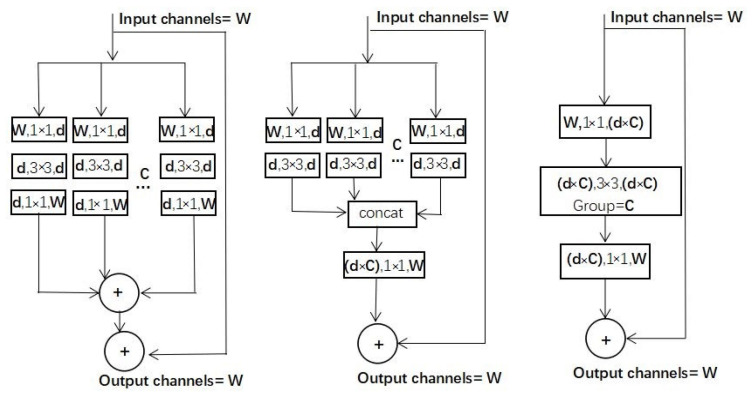
Three forms of ResNeXt [26]. Layers are expressed as (input channels, filter size, and output channels), while C indicates cardinality.

Many practices have proven that deepening or widening the network can improve network performance for convolutional neural networks. Still, these two strategies are also accompanied by an increase in parameters and calculations. ResNeXt [26] combines the advantages of VGG [27] and Inception [28,29,30,31] and applies the unit repetition strategy in VGG to the split-transform-merge strategy in Inception. The cardinality dimension is introduced into the “transform” through the repetition of units that integrate several operations with the same weight. The parameter sharing strategy of these blocks ensures that the complexity of ResNeXt will not increase when the cardinality dimension is increased. Besides, ResNeXt follows the shortcut connection of ResNet [32] to avoid the gradient explosion and gradient disappearance problems that may be encountered in the back-propagation process. Through deformation, ResNeXt has three forms [26], as shown in Figure 5.

**Figure 6:**
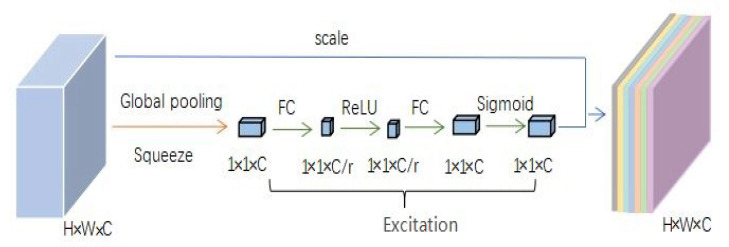
Structure of Squeeze-and-Excitation net (SEnet). The yellow arrow represents the squeeze stage, the blue arrow represents the scale stage, and the green arrow represents the excitation stage.

**Figure 7:**
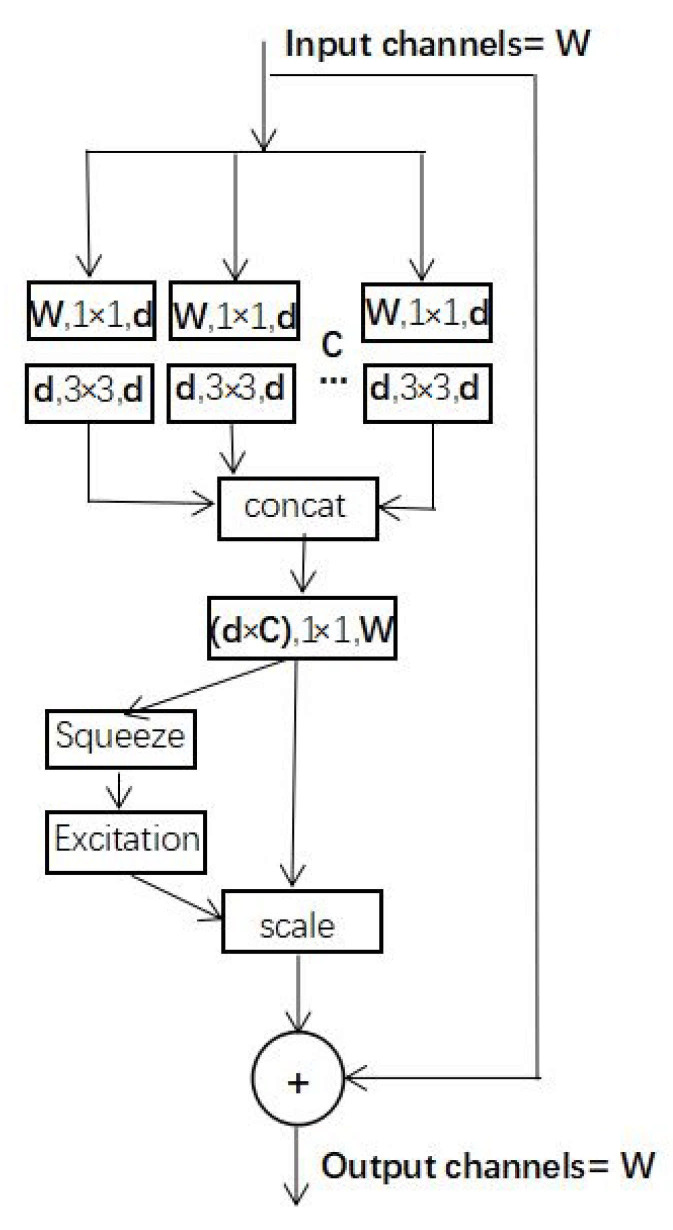
Structure of SE-ResNeXt used in the study. Layers (represented by a rectangular box) are expressed as (input channels, filter size, and output channels). C is cardinality. Squeeze, excitation, and scale are three stages of SEnet.

SEnet (Squeeze-and-Excitation net) [19] can be embedded in other networks as a substructure [33]. The highlight of this network is the introduction of the attention mechanism to the feature channels. SEnet can promote useful features while suppressing useless ones. This network includes the squeeze, excitation, and scale stages. By using global average pooling, squeeze obtains the global distribution of the feature channel. In excitation, two fully connected (FC) layers are used to construct channels’ correlation. This stage outputs importance factors with the same number as input features. Finally, the importance factors obtained from excitation are weighted to the original feature through the scale stage to realize the feature importance calibration. The structure of SEnet is shown in Figure 6. The SE-ResNeXt used in this study placed SEnet before the residual shortcut of ResNeXt. SE-ResNeXt’s structure is shown in Figure 7.

#### 2.2.3. YOLO-S-CIOU Network Structure

Due to the low recall rate of YOLOv3 [14], the study used SRXnet instead of Darknet53 to generate an improved model YOLO-S-CIOU. Here, YOLO, S, and CIOU mean YOLOv3, SRXnet, and CIoU loss, respectively. SRXnet is a newly constructed CNN proposed by this study, which is composed of five groups of nSR modules with n values of 1, 2, 8, 8, and 4, respectively. nSR is formed by the superposition of n SE-ResNeXt [19], which makes SRXnet maintain a large depth and increase the cardinality dimension. SE-ResNeXt is mainly composed of 16 sets of transformations with the same weight, in which each of the transformations is composed of two CBL structures with a convolution kernel of 1 × 1 and 3 × 3. CBL is the most used basic component in the model, including a convolution layer, a batch normalization layer (BN) [29], and a LeakyReLU layer. SEnet is added at the end of each SE-ResNeXt to ensure that the advantages of features learned from the current SE-ResNeXt are preferentially applied to the learning process of the next SE-ResNeXt. The addition of SEnet can restrain the interference of some useless features to learning. Besides, a shortcut connection is still used as a strategy to prevent the gradient problem in backpropagation in SE-ResNeXt.

**Figure 8:**
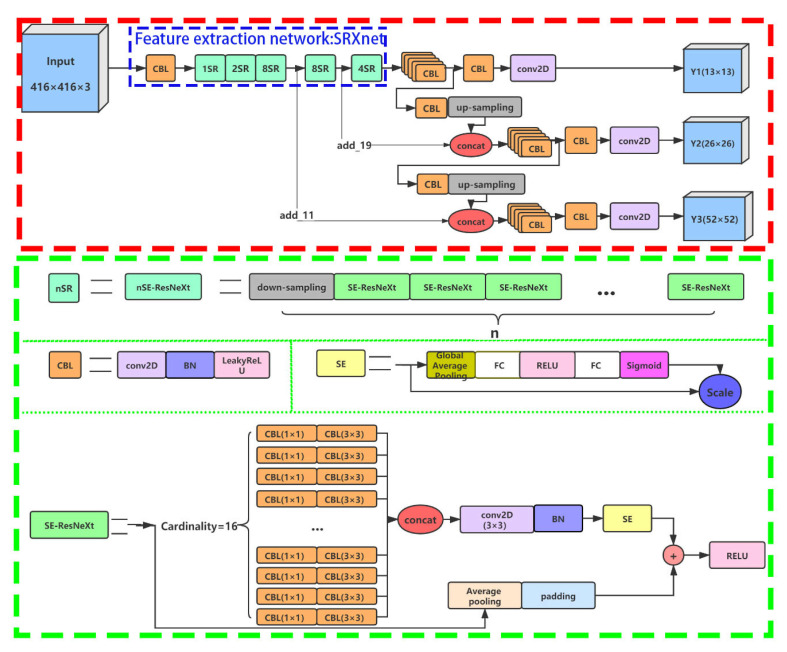
Structure of YOLO-S-CIOU. The part in the red dashed box represents the overall structure of YOLO-S-CIOU; the part in the green dashed box represents the 4 substructures of YOLO-S-CIOU; and the part in the blue dashed box represents SRXnet. Modules with the same color represent the same operation. CBL is a structure containing a convolution layer (conv2D), a batch normalization (BN) layer, and a LeakyReLU layer. Concat represents concatenate.

YOLO-S-CIOU outputs the prediction results on the scales of 13 × 13, 26 × 26, and 52 × 52. The input images of 416 pixels × 416 pixels are downsampling five times in SRXnet and become 13 × 13 feature maps. After post-processing, the prediction frames are obtained on a scale of 13 × 13. By using convolutions with a step size of 2, SRXnet achieved 5 downsampling. Besides, the output of the second 8SE-ResNeXt (add_19) is concatenated with the upsampling result of the first branch to obtain the prediction results on the scale of 26 × 26. The output of the first 8SE-ResNeXt (add_11) was concatenated with the upsampling result of the second branch to obtain the prediction results on the scale of 52 × 52. The detailed structure of YOLO-S-CIOU is shown in Figure 8.

#### 2.2.4. Loss Function

To solve the problem of inaccurate bounding box regression in YOLOv3, the study improved the loss function of YOLO-S-CIOU. The loss function of YOLOv3 primarily consisted of four parts: XY coordinate loss (XY_loss), length and width loss (WH_loss), confidence loss (Confidence_loss), and category loss (Class_loss). XY_loss and WH_loss together constitute the position loss of YOLOv3. The prediction boxes used in the calculation of position loss of YOLOv3 were mainly obtained by regression under the intersection over union (IoU). However, IoU only considers the overlap area between the ground truth box and the prediction box. The optimization direction cannot be given when the two boxes contain one another or do not intersect.

Generalized intersection over union (GIOU) [34] considers the minimum enclosing rectangle of two boxes based on IoU, which solves the optimization direction problem when two boxes do not intersect. However, GIOU solves this problem by expanding the prediction box until it intersects with the ground truth box. When the two boxes intersect, GIOU degenerates into IoU. Therefore, GIOU cannot accurately reflect the overlapping relationship between the two boxes and cannot give the optimization direction when one box is surrounded by another. By minimizing the distance between the ground truth box and the prediction box, distance-IoU Loss (DIOU Loss) [20] solves the problem of IoU and GIOU and greatly improves the speed of regression. DIOU can be calculated using the following equation:

(1) $$DIOU=1-IOU+\frac{l{\left(O_{p},O_{l}\right)}^{2}}{C^{2}}$$

where *O_p_* and *O_l_* are the prediction box’s center point and the ground truth box’s center point, respectively, *l* represents the Euclidean distance between the two center points, and *c* represents the diagonal distance of the minimum enclosing rectangle of two boxes.

DIOU can well realize the regression of prediction box position, but when the center of the prediction box coincides with the center of ground truth box, the optimization stops. Therefore, to achieve a more comprehensive optimization, Complete-IoU Loss (CIoU loss) [20] comprehensively considers the overlap area, the distance of central points, and aspect ratio’s uniformity and adds penalty terms for the shape. CIoU can be calculated using the following equation:

(2) $$CIOU=1-IOU+\frac{l{\left(O_{P},O_{l}\right)}^{2}}{c^{2}}+\alpha \times \upsilon$$

where α is a balance factor, and *υ* is a shape penalty term. The formula is as follows:

(3) $$\upsilon =\frac{4}{\pi^{2}}{\left(arctan\frac{w_{t}}{h_{t}}-arctan\frac{w_{p}}{h_{p}}\right)}^{2}$$

(4) $$\alpha =\frac{\upsilon }{\left(1-IOU\right)\;+\;\upsilon }$$

Previous studies have shown that the regression loss can be directly used as the position loss of YOLOv3. To fully consider the relationship between the ground truth box and the prediction box, YOLO-S-CIOU uses CIoU loss as position loss, which can be calculated using the following equation:

(5) $$CIOU\_LOSS=Confidence\times (2-w\times h)\times \;\left(1-CIOU\right)$$

where *Confidence* is adopted to show whether the target is contained in the predicted box, (2 − *w* × *h*) is the CIoU loss weight of each predicted box.

### 2.3. Evaluation Indicators

The confusion matrix, also known as the error matrix, is a matrix composed of four first-level indicators. The four indicators are false negative (FN), false positive (FP), true negative (TN), and true positive (TP). These indicators, in turn, represent the positive targets that the model classifies as negative, the negative targets that the model classifies as positive, the negative that the model classifies as negative, and the positive targets that the model classifies as positive. The four indicators are relatively basic and do not clearly reflect the comprehensive performance of the model. Recall (R) and precision (P) [35] are generated based on these four basic statistical values. Precision measures the ratio of correct targets in the targets that are judged to be correct. Recall measures the ratio of targets that are judged to be correct in the correct targets. The calculation formulae are as follows:

(6) $$Precision=\frac{TP}{TP+FP}$$

(7) $$Recall=\frac{TP}{TP+FN}$$

Based on the two second-level indicators, the precision–recall curve (PRC) [36] can be generated to visualize the performance of the model. Recall–precision is used as the horizontal coordinate axis and longitudinal coordinate axis, respectively. If the PRC of one model is completely enclosed by the PRC of another model, the performance of the latter tends to be better than that of the former. However, when the two curves intersect, the performance of the model cannot be judged by this standard. At this time, the F1 score, a further third-level index, is typically used. The larger the F1, the greater the model’s performance. F1 is calculated using the following equation:

(8) $$F1=\frac{2\times Precision\times Recall}{Precision+Recall}$$

The average precision (AP) [37] is an indicator commonly used in regression problems. AP equals to the area surrounded by model’s PRC and coordinate axes, which can be used to calculate the model’s quality in one category.

### 2.4. Implement Environment and Model Training

The experiment was carried out using TensorFlow and Keras on a platform equipped with the operating system Windows 10, an RTX2080Ti graphics (11 GB), and an Intel i9-9900k processor. The configurations were progressively optimized during the training phase. Finally, an Adam optimizer was used. They used an Adam optimizer. The size of each batch was 8. The initial rate of learning was 10^−3^. The learning rate decreased 10 times if the validation loss stayed unchanged within 20 epochs. If validation loss did not change within 50 epochs, training would end prematurely. The models were all trained for 500 epochs, but some of them stopped early.

## 3. Results and Discussion

### 3.1. Index Evaluation

**Figure 9:**
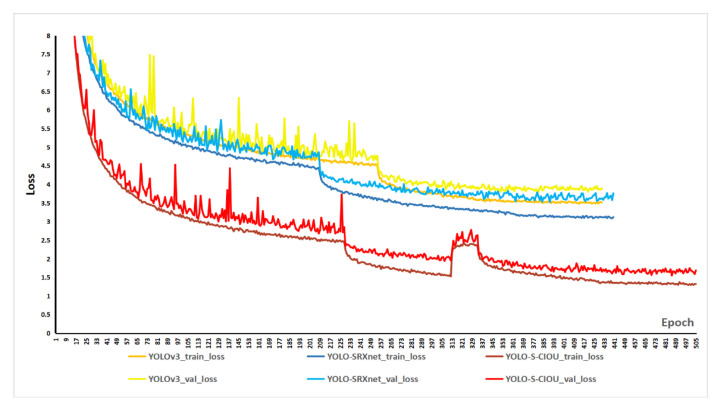
Loss curves of three networks. The two red-tone curves are the training and validation loss curves of YOLO-S-CIOU; the two blue-tone curves are the training and validation loss curves of YOLO-SRXnet; and the two yellow-tone curves are the training and validation loss curve of YOLOv3. The more vivid one in the two curves of the same network is the validation loss curve.

The method proposed in this study improved the feature extraction network and loss function of YOLOv3. First, we used SRXnet instead of Darknet53 as a feature extraction network. Second, a more effective CIoU loss was substituted for the position loss of YOLOv3. To test the impact of these two improvements on the detection performance of the CNN, we trained and tested three networks: the original YOLOv3, YOLO-SRXnet that introduces the first improvement, and YOLO-S-CIOU that introduces both improvements. Used as a cycle were 500 epochs. Three networks’ training and validation loss are displayed in Figure 9. There are six curves in total, consisting of three training loss curves and three validation loss curves. For the convenience of comparison, the training loss curve and the validation loss curve of the same network are displayed in similar colors. Still, the color of the validation loss curve is more vivid. Through analysis, the following two conclusions can be drawn:

- All the training and validation loss curves were reasonable, which indicates that all three networks performed well on the dataset.
- With the same loss function, the YOLO-SRXnet shows a lower training and validation loss value than YOLOv3, which indicates better performance than the traditional YOLOv3 network.

In addition to the loss curve, the precision (P), recall rate (R), F1 score, and average precision (AP) were also used to evaluate the three networks quantitatively. Table 2 presents the specific values. The values of P and R are the corresponding results when F1 was maximized. The F1 and AP of the YOLO-S-CIOU were 97.5% and 97.62%, respectively. Compared with YOLOv3, these two indicators increased by 0.50% and 2.23%, respectively, and the parameters of YOLO-S-CIOU and YOLO-SRXnet (59,065,366) were reduced by 2,510,977 (about 4%). From the F1 and AP of the three networks, it was observed that the improvement of feature extraction network had made a major contribution to the improvement of model indexes and the decrease of parameter quantity, which achieved a 0.5% increase in F1, 2.1% increase in AP and 2,510,977 (about 4%) decrease in parameter quantity. This fully proves the effectiveness of increasing the dimension of cardinality and the use of the SEnet structure. The introduction of CIoU loss in the loss function also increased the AP value by 0.13%, indicating that the regression of the bounding box was more accurate with CIoU. Additionally, it can better guide the learning process of the model in the backpropagation.

**Figure 10:**
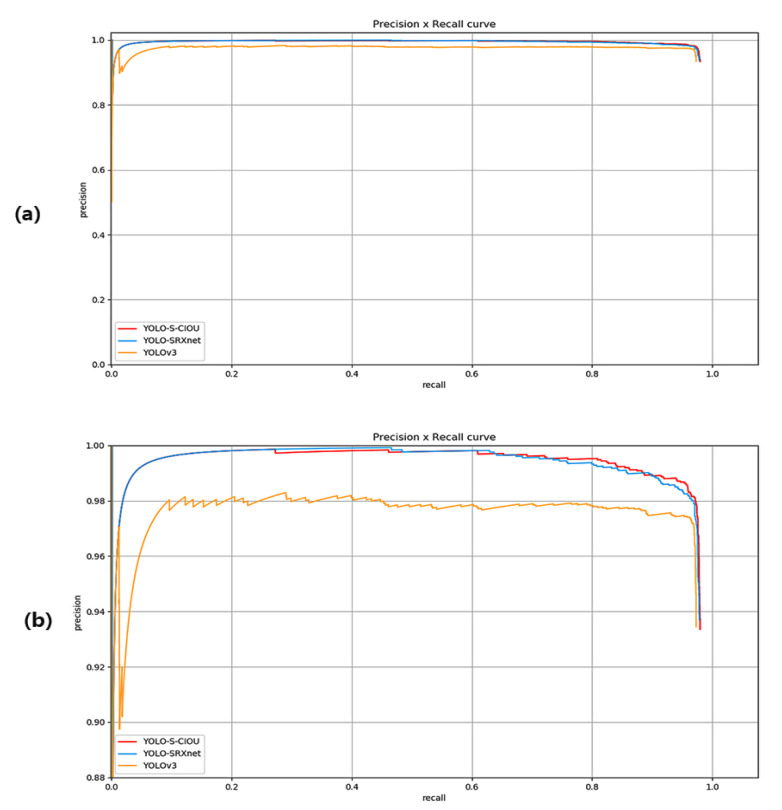
Three networks’ precision–recall curves (PRC). (**b**) is a PRC enlarged from (**a**) to better reflect the relationship of the three curves.

To evaluate the networks vividly and intuitively, Figure 10 displays the three networks’ precision–recall curve (PRC). According to the characteristics of PRC, it was observed that the network represented by the latter curve is better when one curve is enclosed by another. In the figure, it can be clearly seen that the curves of YOLO-SRXnet and YOLO-S-CIOU surrounded the curve of YOLOv3. Although the curves of YOLO-SRXnet and YOLO-S-CIOU partially overlapped, the fact that the curve of YOLO-S-CIOU surrounded that of YOLO-SRXnet is still clearly shown. Therefore, YOLO-S-CIOU achieved the most effective in these three networks, followed by YOLO-SRXnet.

As can be seen from F1, AP, and PRC, SRXnet significantly improved network performance. Moreover, the performance improvement would not increase the parameters’ number and complicate the network. Compared with Darknet53 in YOLOv3, SRXnet increased the cardinality dimension, but the depth and channel number almost remained unchanged. The increase of the cardinality dimension led to more transformation operations, which further deepened the learning level. However, parameter sharing in the operation avoided the increase of parameter quantity. This proved that the use of cardinality dimension was more valuable than the networks’ depth and width in practical applications with restricted hardware conditions. The attention mechanism introduced by SEnet in the network could make the network acquire the importance of features while learning the features of the target. The importance further helped the deep learning of features in bringing the advantages of feature learned into full play and further improved network’s learning efficiency. Besides, CIoU could further optimize the prediction box based on IoU, so adding CIoU to the loss function can further improve the accuracy of the model.

### 3.2. Application Evaluation

**Figure 11:**
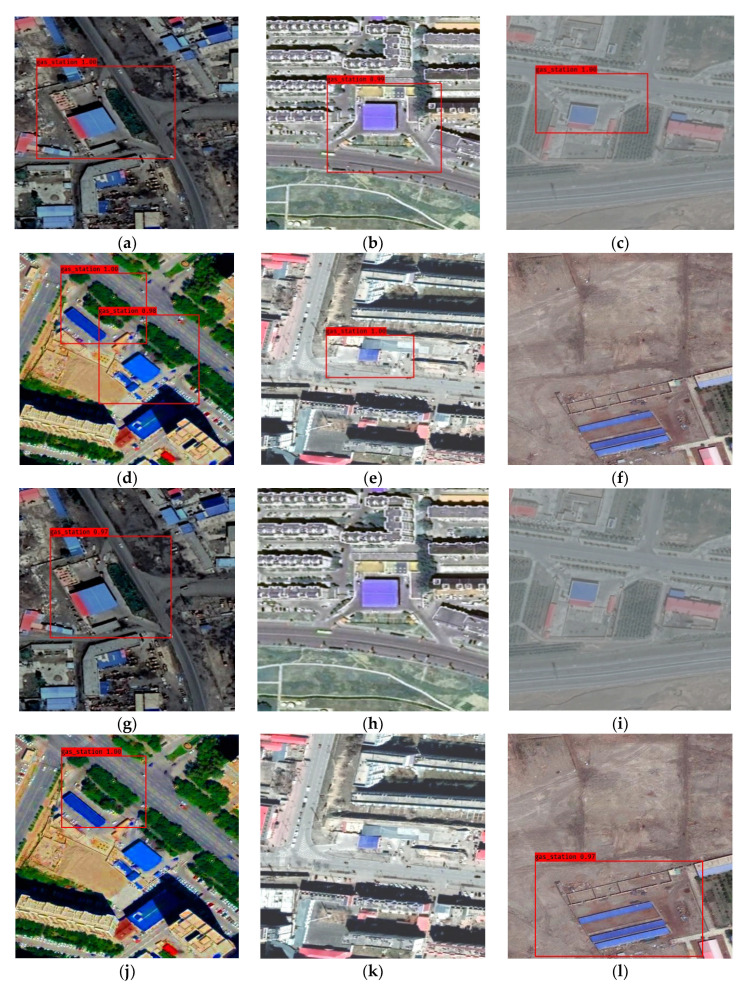
Test results of YOLO-S-CIOU and YOLOv3 on 416 pixels × 416 pixels gas station pictures with different characteristics. (**a**–**f**) are the test results of YOLO-S-CIOU and (**g**–**l**) are the test results of YOLOv3.

Here, we would evaluate the detection performance of YOLO-S-CIOU and YOLOv3 on 416 pixels × 416 pixels images and remote sensing images. Figure 11 showed the detection results of YOLO-S-CIOU and YOLOv3 on six 416 pixels × 416 pixels gas station images, each with different qualities. Figure 11a–f showed the detection results of YOLO-S-CIOU, while Figure 11g–l showed the detection results of YOLOv3. By comparing same images’ detection results, it was observed that the gas stations with clear characteristics could be accurately detected by both networks, as shown in Figure 11a,g. However, when the image color was abnormal (Figure 11b,c,h,i), multiple targets were adjacent (Figure 11d,j), and the image features were not obvious (Figure 11e,k), the missing detection phenomena of YOLOv3 were serious. Additionally, YOLOv3 also had serious false detection phenomena (Figure 11l). YOLO-S-CIOU avoided these errors perfectly. Therefore, compared with YOLOv3, YOLO-S-CIOU was more robust and had stronger detection ability and a higher precision for images of different quality.

**Figure 12:**
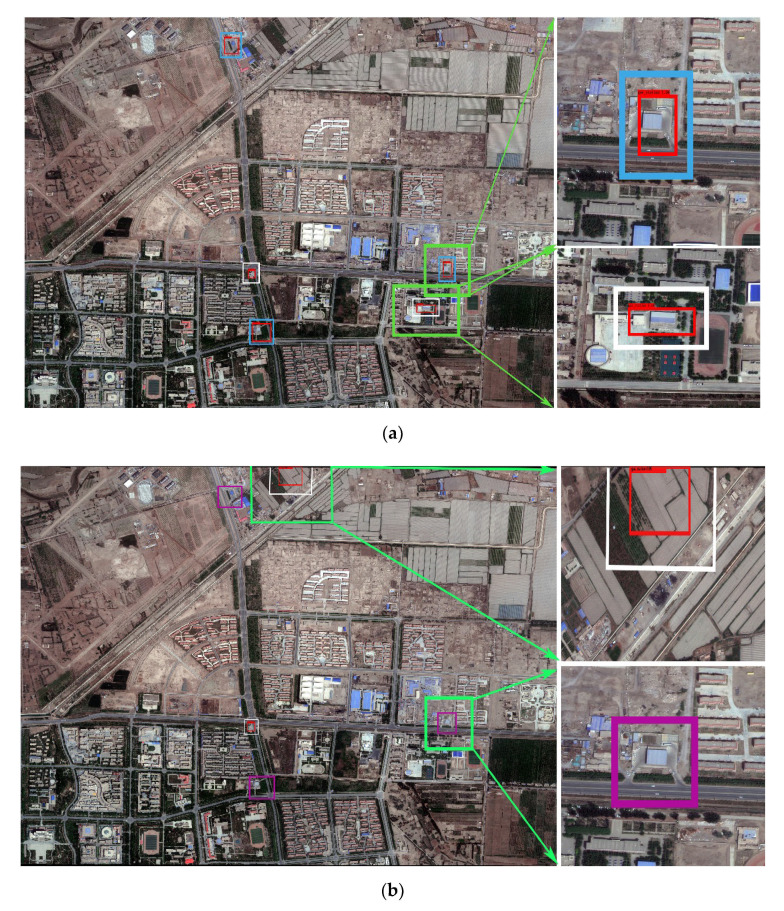
Detection results of gas stations in remote sensing images by YOLO-S-CIOU and YOLOv3. (**a**) is the detection result of YOLO-S-CIOU on WorldView data and (**b**) is the detection result of YOLOv3 on WorldView data. A red box in (**a**) and (**b**) is a detection result box by networks; a blue box in (**a**), (**b**) refers to true positive (TP); a purple box refers to false negative (FN); a white box refers to false positive (FP), and a green box in (**a**) and (**b**) indicates the area of the enlarged images.

It was observed that the detection effect of YOLO-S-CIOU on 416 pixels × 416 pixels images was especially excellent, and its accuracy was significantly higher than that of YOLOv3. To test the usability of YOLO-S-CIOU in the remote sensing field and further reflect this method’s advantages, we used YOLOv3 and YOLO-S-CIOU to experiment with remote sensing images introduced in Section 2.1.3. Figure 12 shows the detection results of gas stations in remote sensing images by the two networks. By comparing the location of the gas stations detected in the remote sensing images (represented by the red box in Figure 12) with the actual location (represented by the yellow boxes and points in Figure 3), it could be seen that although YOLO-S-CIOU had some missing and false detection in the face of more complex remote sensing images, the error phenomenon was obviously lighter than that of YOLOv3. More than half of the gas stations could be detected. This fully proved that compared with the original YOLOv3, YOLO-S-CIOU was more competent for detecting specific buildings in remote sensing images.

**Figure 13:**
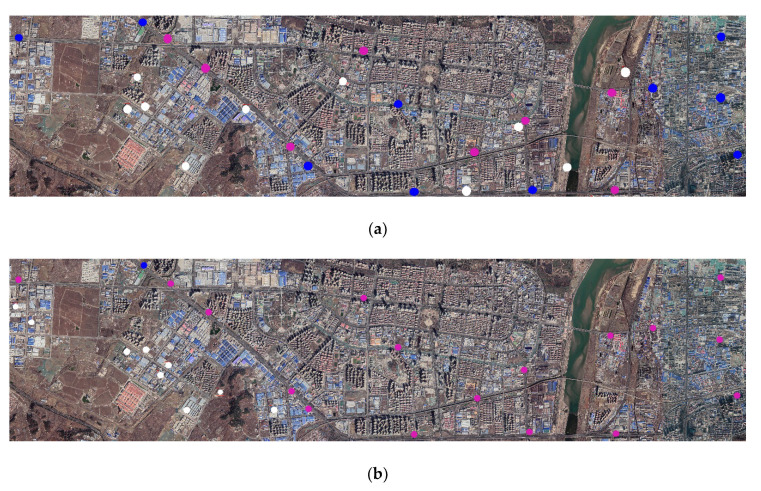
Detection results of gas stations in remote sensing images by YOLO-S-CIOU and YOLOv3. (**a**) is the detection result of YOLO-S-CIOU on Google maps and (**b**) is the detection result of YOLOv3 on Google maps. A blue point in (**a**), (**b**) refers to the true positive (TP); a purple point refers to the false negative (FN); and a white point refers to the false positive (FP).

Although the images of Tumshuk City did not participate in the network training, Tumshuk City was adjacent to the geographical location of the data source of the training data. To further test the robustness and universality of YOLO-S-CIOU, we also used Google maps of Yantai City to test the effect of the improved model. Figure 13 displays the outcome of identification. It could be seen that although the regional characteristics of Yantai City and Xinjiang Uygur Autonomous Region were completely different, YOLO-S-CIOU still had a good detection effect for Yantai City. Although there were some errors, the problems were obviously less than that of YOLOv3. The detection results fully proved that YOLO-S-CIOU was also robust to changes in the study area.

To quantitatively evaluate the detection effect for Tumshuk City and Yantai City by the two networks, this study conducted statistics based on Figure 12 and Figure 13 and obtained Table 3. For WorldView data of Tumshuk City, the recall (R) and precision (P) of YOLOv3 were 0% and 0%, respectively, and the R and P of YOLO-S-CIOU were 100% and 60%, respectively. For the Google maps of Yantai City, the R and P of YOLOv3 were 5.6% and 10%, respectively, and the R and P of YOLO-S-CIOU were 55.6% and 50%, respectively. The R and P of YOLO-S-CIOU were 50% and 40% higher than those of YOLOv3, respectively. These indicators further proved that YOLO-S-CIOU was more robust for remote sensing images in different regions and showed that the improved model could improve the low recall rate of YOLOv3.

The detection results in remote sensing images exposed some problems, such as false detection, missing detection, and the change of P and R values of detection results when changing the detection area. First, this was likely due to the complex backgrounds and a wider variety and number of ground objects in the remote sensing images, which resulted in interference; second, although some data augmentation strategies were used before training, the diversity of the training data was still relatively weak. The existing problems would be solved gradually by expanding the dataset. Despite these problems, the proposed CNN was still greatly improved compared with the conventional approach. The problems of missing and false detection were also obviously lighter than these of the original YOLOv3. Although the P and R values of remote sensing images in Yantai City obtained by YOLO-S-CIOU were smaller than the two indicators of Tumshuk City, they were still significantly higher than the corresponding detection results of YOLOv3. Therefore, YOLO-S-CIOU could be more competent for the detection of specific buildings in remote sensing images.

## 4. Conclusions

In this study, we developed a novel CNN model named YOLO-S-CIOU that was improved based on YOLOv3 to detect specific buildings in remote sensing images. The improvement mainly included two parts: the feature extraction network’s improvement and the loss function’s improvement. In the first part, module Darknet53 in YOLOv3 was replaced with SRXnet to significantly improve the feature learning ability of YOLO-S-CIOU while maintaining a similar complexity as YOLOv3. In the second part, CIoU Loss was used as position loss in the loss function to obtain a better regression for the bounding box. We took gas stations as an example. GS dataset was used to perform the experiment. Results showed that YOLO-S-CIOU achieved an AP of 97.62%, an F1 score of 97.50%, and had 59,065,366 parameters. Compared with YOLOv3, YOLO-S-CIOU had 2,510,977 (about 4%) fewer parameters, and it improved AP by 2.23% and the F1 score by 0.5%. Besides, YOLO-S-CIOU avoided many errors of YOLOv3 regarding the detection of 416 pixels × 416 pixels images and obtained a higher P and R values in the remote sensing images detection. The detection results of different regions showed the strong robustness of YOLO-S-CIOU and showed the effectiveness of this model in specific buildings’ identification in remote sensing images. However, the detection results also exposed some problems such as false detection, missing detection, and the change of P and R values when changing the detection region. These problems might be overcome by expanding and optimizing the GS dataset, which would be the next direction of work. The detection effects of YOLO-S-CIOU on other types of buildings in remote sensing images would also be tested in future research.

## Figures and Tables

**Table 1:** The dataset division.

	Training Set	Validation Set	Testing Set
Gas station	8400	2100	2520

**Table 2:** Specific index values of three networks.

	P (%)	R (%)	F1 (%)	AP (%)	Parameter Size
YOLOv3	97	97	97	95.39	61,576,343
YOLO-SRXnet	97	98	97.50	97.49	59,065,366
YOLO-S-CIOU	98	97	97.50	97.62	59,065,366

**Table 3:** Comparison of detection performance of YOLOv3 and YOLO-S-CIOU on remote sensing images. TP, FN, and FP are calculated according to the number of various points and boxes in Figure 12 and Figure 13, and P and R are calculated according to the formula in Section 2.3.

Images	Model	TP	FP	TN	FN	P (%)	R (%)
WorldView data of Tumshuk City	YOLO-S-CIOU	3	2	-	0	60	100
	YOLOv3	0	2	-	3	0	0
Google map of Yantai City	YOLO-S-CIOU	10	10	-	8	50	55.6
	YOLOv3	1	9	-	17	10	5.6

## Data Availability

Not applicable.
